# Reevaluating Anomalous Electric Fields at the Air–Water Interface: A Surface-Specific Spectroscopic Survey

**DOI:** 10.1021/jacs.5c14541

**Published:** 2025-12-03

**Authors:** Joseph C. Shirley, Zi Xuan Ng, Kuo-Yang Chiang, Yuki Nagata, Yair Litman, Arsh S. Hazrah, Mischa Bonn

**Affiliations:** 28308 Max Planck Institute for Polymer Research, Ackermannweg 10, Mainz 55128, Germany

## Abstract

The notion that large electric fields at the air–water interface catalyze spontaneous chemical reactions has sparked significant debate, with far reaching implications for atmospheric chemistry and interfacial reactivity. Using vibrational sum frequency generation (SFG) spectroscopy, we test this hypothesis within the framework of this surface-specific method, comparing local electric field strengths at the air–water interface and in bulk water. By applying established vibrational frequency-to-field mappings to the OH stretch of interfacial and bulk water, we extract effective electric field distributions under ambient conditions. Contrary to prevailing claims, our SFG results reveal no spectroscopic evidence within this method for exceptionally strong or long-lived interfacial electric fields. Instead, bulk water consistently exhibits broader field distributions. The absence of key spectral signatures, such as red-shifted continua, or slowed spectral diffusion, further undermines the idea of anomalous surface fields. Our findings suggest that exceptionally large, long-lived interfacial fields are unlikely. This calls into question interpretations that attribute droplet chemistry primarily to such electric fields.

## Graphical abstract

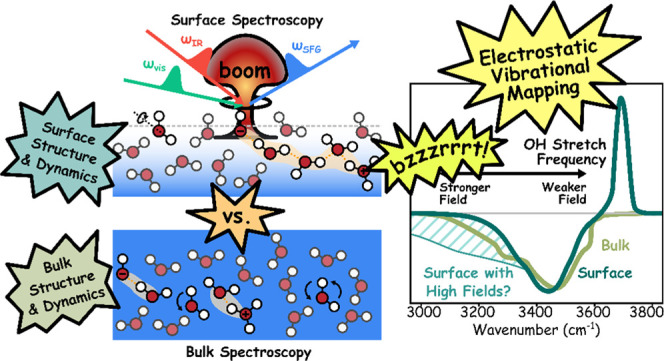

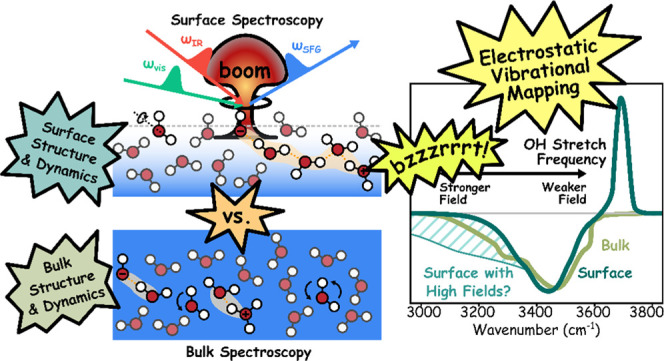

## Introduction

Interfaces between gas and liquid, liquid and liquid, or solid and liquid are chemically distinct environments, where reactions unfold with unique dynamics and selectivity absent in the bulk.^1–5^ Interfaces provide unique physicochemical environments characterized by broken symmetry,^6^ altered solvation,^7^ and depth-dependent ion distributions,^8^ all of which can influence molecular orientation,^9,10^ reaction pathways,^11,12^ and other interfacial properties.^13,14^

The gas–liquid interface, especially the air–water boundary, has been the focus of intense investigation due to its pivotal role in both natural and engineered systems. In atmospheric chemistry, reactions at aqueous aerosol or cloud droplet interfaces are central to processes like ozone degradation,^15^ sulfate formation,^16^ and organic aerosol aging.^17^ Remarkably, this interface has also been implicated in atypical reaction pathways, including the spontaneous generation of hydrogen peroxide (H_2_O_2_) in pure water microdroplets, a phenomenon attributed to water autoionization coupled with interfacial effects.^18,19^ However, subsequent studies have shown that H_2_O_2_ formation is strongly influenced by dissolved oxygen and water–solid interfaces, rather than arising at pristine air–water boundaries.^20–25^ Observations of accelerated reaction rates and unconventional product formation in aqueous microdroplets, emulsions, and sprays have led to growing speculation that interfaces may catalyze reactions even without added reagents or external catalysts.^26,27^ Yet recent critical analyses have highlighted that artifacts or secondary processes can mimic such reactivity, calling into question the extent to which interfacial electric fields are responsible. In parallel, alternative explanations such as dissolved-oxygen chemistry and droplet charging have been advanced.^28–32^ Some recent reports have also suggested that interfacial curvature can enhance local electric fields, thereby influencing reactivity in charged water microdroplets.^33^ These claims, if substantiated, carry profound implications as they redefine the role of interfaces in environmental and synthetic chemistry. Crucially, many interpretations of these findings rest on the specific hypothesis that large or long-lived electric fields exist at the neat air–water interface, providing an electrostatic driving force capable of stabilizing transition states or polarizing bonds.^34,35^ This distinguishes such claims from other known interfacial enhancements that arise from interfacial changes in solvation structure,^36^ rather than electrostatic effects.

In this study, we critically evaluate the hypothesis of large, sustained interfacial electric fields at the neat air–water interface. Using vibrational sum-frequency generation (SFG) spectroscopy, we take advantage of the fact that the OH stretch vibration of interfacial water is a direct reporter of the local electric field through well-established frequency-to-field relationships. This approach allows us to quantitatively map interfacial field strengths from the measured vibrational spectra themselves. If strong electric fields (on the order of 100–1000 MV/cm)^37^ were present in a way atypical of bulk water, they would produce characteristic perturbations of the OH stretch band such as broad continua, pronounced redshifts, and increased anharmonicities, arising from field-induced changes in water’s hydrogen-bonding structure. However, the observed SFG spectra reveal only features consistent with a modestly polarized hydrogen-bonded network, with no signatures of such large fields. Applying the same vibrational frequency-to-field mapping to both interfacial and bulk water responses further shows that large electric fields are statistically more probable in bulk water than at the interface, contradicting assertions that anomalously strong interfacial fields arise and drive unusual behavior at neat interfaces.

## Results and Discussion

### Defining Interfacial Electric Fields

To assess the hypothesis of field-driven chemistry at aqueous interfaces, we first define what is meant by “electric field” in this context. The concept spans a range of physical scenarios that are not mutually exclusive (certain types of fields can give rise to others) yet each can lead to distinct spectroscopic and chemical consequences. In all of the following scenarios, the line between applied fields, local fields along a bond vector, and non-Coulomb effects approximated by effective “fields” is blurred. Even with known Stark probes, local solvation effects require careful calibration and can make generalization challenging.^38^ In this work, we will operate under the assumption that water is the least perturbative probe molecule of the neat air–water interface and also that the local environment of water is the most relevant to characterize for water-as-a-reagent chemistry. Specifically, we consider:

1. Static E-fields: A static or equilibrium electric field, often considered as normal to the interface, arising from net charge separation (e.g., surface ion gradients) or collective dipolar alignment of water molecules.
2. Field distributions: A distribution of local “fields” experienced by O–H bonds due to heterogeneous solvation environments and hydrogen-bond fluctuations, some of which may mimic field-like effects.
3. Fluctuations: A temporal overlap between field-like effects and molecular bond orientations, yielding chemically relevant perturbations. For example, having slower solvent or field fluctuations could enhance coupling to reactive coordinates.

While these scenarios offer plausible mechanisms for interfacial electric fields to influence chemical reactivity, the central hypothesis, that such fields are sufficiently large and persistent to drive reactions, has been met with substantial critique. Specifically, challenges have been raised against both the underlying claim that interfacial electric fields are stronger than those in bulk water, and the broader conclusion that such fields, even if present, are capable of inducing chemical transformations.^39,40^ These critiques highlight the need to distinguish between different definitions of field magnitude, spatial extent, and time scale, and to assess their spectroscopic consequences using methods capable of resolving molecular-level behavior. In the following sections, we examine the spectroscopic signatures associated with each of these proposed field scenarios to determine whether comparatively large fields or slower field fluctuations at the air–water interface are consistent with experimental observations.

### Vibrational Sum-Frequency Generation Spectroscopy

To test whether large electric fields are present at the air–water interface, we primarily employ vibrational SFG spectroscopy, a surface-specific technique that directly probes the orientation and hydrogen-bonding environment of interfacial water molecules. The surface sensitivity of SFG spectroscopy arises from the selection rules governing second-order nonlinear optical processes: in isotropic, centrosymmetric media like bulk water, the second-order susceptibility χ^(2)^ vanishes, ensuring that only interfacial molecules contribute to the signal.^41^ In addition, χ^(3)^ contributions measured with the SFG technique provide a bulk analogue for a direct comparison with the surface χ^(2)^ response.^42^

The local environment sensitivity of SFG is conferred through the potential energy well of the molecule of interest. Generally, oscillators are anharmonic, and vibrational energy levels can be envisioned as lying within an asymmetric potential. This well-established framework describes both the spacing between vibrational states and the bond dissociation energy. Perturbations such as hydrogen bonding or electric fields modulate the curvature and depth of this potential, thereby shifting the energy of the fundamental (0 → 1) vibrational transition.^38^ Bond weakening, whether due to strong hydrogen bonding or the influence of an electric field, typically leads to a redshift in the vibrational frequency. Therefore, the OH stretch serves as an intrinsic reporter of the local field and bonding environment. While Stark shift spectroscopy of nonwater probe molecules has been used to probe interfacial fields, it is more straightforward to use field-induced Stark shifts of the OH groups themselves.^38^

Thus, SFG provides an experimental framework that allows direct comparison between the energy states of water molecules at the surface and in the bulk. The approach is schematically summarized in Figure 1. In particular, the shaded region in Figure 1B denotes the red-shifted continuum expected under conditions of comparatively strong static interfacial electric fields, which provides the conceptual basis for distinguishing bulk and interfacial responses.^43^ We note that we study extended, planar surfaces, rather than micron-sized droplets, which is justified by the negligible curvature of micron-sized droplets on molecular length scales.

**Figure 1:**
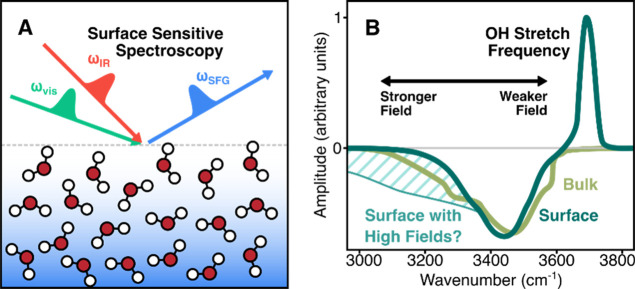
(A) Schematic of vibrational sum frequency generation spectroscopy, which selectively probes the air–water interface through its sensitivity to noncentrosymmetric regions. (B) Sketch comparing the expected OH stretch SFG responses of bulk and interfacial water. The shaded region highlights a red-shifted continuum that would be expected under conditions of comparatively strong static interfacial electric fields.

### Mapping Vibrational Frequencies to Local Fields

While SFG provides access to frequency shifts of the OH stretch vibration, which are connected to electric fields, translating these shifts into electric field strengths quantitatively requires an appropriate model. In this context, vibrational maps serve as a crucial bridge. These empirically derived relationships connect molecular dynamics outputs, such as the instantaneous electric field or electrostatic potential experienced by a molecular bond, to the energy of its vibrational transitions. Accordingly, OH stretch frequency shifts provide a sensitive and quantitative measure of the local interfacial electric field that shapes molecular structure and reactivity. Here, they allow linking experimental observables to the underlying molecular-scale electrostatics.^38^ In water, this mapping is particularly powerful because OH stretch frequencies respond strongly to electric fields, both through direct Stark perturbations and through their tight coupling to hydrogen bonding. A hydrogen bond acceptor not only carries a partial negative charge, generating a local field, but also weakens the OH bond of the donor.^44^ For water, therefore, it bears repeating that the “electric field” sensed by the OH stretch is not purely electrostatic; the non-Condon effects and other nuclear-coordinate-dependent factors make the inferred field an effective rather than a strictly physical quantity.^44–46^ Nevertheless, determining an effective electric field provides a useful comparison with other works that gauge the likelihood of chemical reactivity based upon it. And, given the strong field sensitivity of the OH and OD stretches, vibrational maps have become an essential tool for estimating electric field distributions along hydrogen bonds in aqueous systems.^44,45,47–49^ To be specific, the electric fields involved in this work are (1) projected along the OH bond vector, (2) based on point charges in classical molecular dynamics, (3) exclude the point charges of the molecule of interest. Regarding point 1, we want to emphasize that one does not sample the local electric field over the entire phase space, but only the projected electric field near the hydrogen atoms. Therefore, while the absolute values of fields are model-dependent, they quantitatively link electric fields from computation to vibrational spectra—including IR, Raman, and SFG—with excellent agreement.^38,44,50–52^ This validation provides confidence in the inverse mapping used here, particularly in identifying trends between the interface and the bulk. In this regard, it is also important to note that the distinction between macroscopic, local, and molecular fields has been clarified in recent theoretical work by Becker, Loche, and Netz,^53^ who showed that the apparent discrepancies between density-functional and classical force-field models arise primarily from quadrupolar contributions to the macroscopic surface potential, while the local dipolar field orientations remain comparable.

By applying frequency-to-field mappings to measured spectra, we can estimate the effective electric field experienced by interfacial water molecules. Specifically, we compare the χ^(2)^ response of the air–water interface with the χ^(3)^ bulk response, providing a direct measure of field strength distributions in each environment. The χ^(2)^ signal originates from the noncentrosymmetric interfacial region, which extends approximately 1 nm into the liquid, while the χ^(3)^ spectrum reflects bulk response under externally applied fields, which was obtained by subtracting two experimentally measured Im χ^(2)^ spectra obtained with phase-resolved SFG spectroscopy.^11,42,54,55^ We compare the χ^(3)^ bulk response with the bulk response inferred from the √(α_IR_ *I*_Raman_) spectrum, where α_IR_ and *I*_Raman_ are the bulk infrared and unpolarized Raman signals, respectively. We use √(α_IR_ *I*_Raman_) as a bulk reference because the χ^(2)^ and χ^(3)^ responses obtained via SFG spectroscopy arise from the product of IR and Raman transitions. Previous studies have shown that this quantity closely reproduces the bulk χ^(3)^ spectrum at charged interfaces.^42,56,57^ To minimize spectral complications from vibrational coupling in neat H_2_O or D_2_O, we analyze the OH stretch of isotopically diluted HOD in D_2_O (Figure 2A).^11,42^ All measurements were performed in the *ssp* polarization combination, followed by Fresnel factor corrections to obtain the *yyz* laboratory frame (see Methods and Supporting Information for a more detailed description of polarization and Fresnel factors). This *yyz* frame emphasizes OH groups with a perpendicular component but also includes contributions from OH groups at different tilt angles. Given the intrinsic heterogeneity of the air–water interface, such orientational averaging is physically appropriate and ensures that the extracted field distributions represent the true ensemble of interfacial OH groups. Applying the same polarization geometry to both bulk and interfacial spectra makes these comparisons internally consistent. However, nonperpendicular electric fields may also be of interest to the community, and recent polarization-resolved experiments and simulations further support this approach by showing that distinct OH orientations experience different local fields and vibrational shifts.^58^ The imaginary component of the χ^(2)^ spectrum reveals two distinct bands: a sharp positive peak near 3700 cm^–1^ originating from the “free OH” groups at the surface, representing the topmost layer of the surface, and a broad negative band centered around 3400 cm^–1^ from hydrogen-bonded OH groups pointing into the bulk. The χ^(3)^ spectrum shows a similarly broad feature corresponding to the bonded OH population in the bulk phase. The imaginary component of χ^(2)^ directly reveals interfacial dipole orientation and hydrogen-bonding character, while the χ^(3)^ spectrum was extracted from bulk-sensitive difference measurements under applied field conditions (see Methods). The α_IR_ and *I*_Raman_ spectra were recorded using commercially available instruments as described in the Methods.

**Figure 2:**
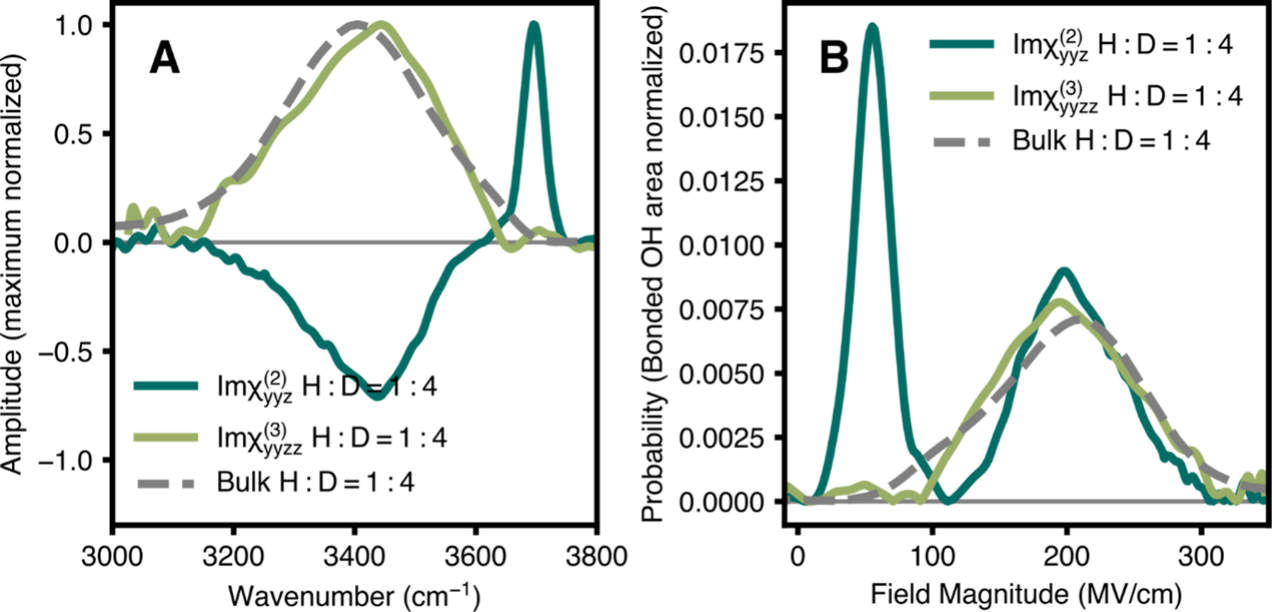
A comparison of bulk vs interfacial field magnitudes. (A) Imχ^(2)^_*yyz*_ spectrum of the OH stretch in isotopically diluted HDO in D_2_O at the air–water interface (dark green),^59^ compared with the respective χ^(3)^ spectrum (light green) and √(α_IR_ *I*_Raman_) spectrum (dotted line), both representing the bulk response. All spectra are normalized to their respective maximum intensities. (B) Probability distribution of electric field strengths projected along the OH bond vector, inferred from the χ^(2)^, χ^(3)^, and bulk √(α_IR_ *I*_Raman_) spectra using established vibrational maps.^60^ Distributions are normalized to the bonded OH area of the bulk. The free OH or topmost layer of the air–water interface experiences relatively small fields, while the OH bonded regions are comparable to those of the bulk. The first moments of the bonded region of the χ^(2)^ spectrum, χ^(3)^ spectrum, and √(α_IR_ *I*_Raman_) spectrum are all 2.1×10^2^ MV/cm.

Using established vibrational maps, we apply the field-to-frequency relationship in reverse to extract the relative probability distribution of electric field strengths along the OH bond from the χ^(2)^, χ^(3)^, and √(α_IR_ *I*_Raman_) spectra (after accounting for relative differences in the response at different frequencies).^60–62^ By performing this calculation, one can determine the relative probability of different electric fields along the OH vector in the bulk or at the interface. The electric fields in Figure 2B are shown as absolute values. Therefore note the direction of the free OH and bonded OH are opposite in sign. This framework can also be generalized to other interfaces, providing a broadly applicable approach for quantifying local field distributions from vibrational spectra. However, an important note about this transformation is that measured spectra do not represent static, purely inhomogeneous lineshapes, but include homogeneous broadening. For the bonded OH and bulk water, inhomogeneous broadening is known to dominate the line shape,^62,63^ as further demonstrated by our estimate in the Supporting Information (Note S3), which shows that approximately 95% of the total line width arises from inhomogeneous effects. Ultimately, these results are normalized for the bulk (bonded OH) population and show that the largest electric fields are more likely to occur in bulk water than at the interface (Figure 2B). The first moments of the bonded region of the χ^(2)^ spectrum, χ^(3)^ spectrum, and √(α_IR_ *I*_Raman_) spectrum are all 2.1×10^2^ MV/cm. These values, while seemingly large, are in fact consistent with the intrinsic local electric fields expected in condensed phases. Kathmann and co-workers have shown that fields of comparable magnitude naturally arise at metal and oxide interfaces due to the organization of electron density and short-range electrostatics.^64^ Molecular dynamics simulations consistently report field strengths in the range of 100–300 MV/cm.^65,66^ This directly challenges claims that unusually strong electric fields exist at the air–water interface. If spontaneous water dissociation were driven by field magnitude alone, such reactions would be expected to occur preferentially in the bulk, not at the surface, given the stronger fields in the bulk. Moreover, the broader distribution of field strengths in the bulk suggests a greater diversity or potentially longer-lived energetic states than those accessible at the interface, depending on the source of the broadening. Together, these findings undermine the idea of anomalous surface field distribution (point 2, distributions). There is also no spectral feature (single peak or overall shift) indicative of a narrowly distributed large field (point 1, static field).

These interpretations are important to contextualize in relation to previous studies estimating field strengths, which found stronger fields at interfaces. Specifically, Zare and co-workers used vibrational Stark shifts of a Rhodamine 800 dye in the bulk and interface of water-in-oil droplets.^67^ Their interpretation of the results was an approximately 10 MV/cm enhancement in the field at the oil–water interface. In computational work by Head-Gordon and others, they find a similar 16 MV/cm increase in field at the interface, specifically for the free OH at the air–water interface.^37^ These calculations were performed using *ab initio*molecular dynamics and report more on the “mean inner potential” rather than the field by external charges provided in this work. In the present work, we agree that the projection of the electric field on free OH is oppositely oriented with respect to bonded OH and on the order of 10–100 MV/cm. However, we interpret that the proximity of the free OH energy to that of the gas phase OH stretch must imply a smaller perturbation to the potential energy surface than the bonded OH or bulk.

If comparatively static strong interfacial electric fields were present at the interface, one would expect a dramatic alignment of water molecules^68^ and enhanced water dissociation,^69^ both of which would significantly perturb the SFG spectra.^70^ Let us consider in more detail the case of electrolyte solutions containing ions that strongly interact with water. Most inorganic ions lack intrinsic surface activity and preferentially remain solvated in the bulk, as shown by both experimental measurements and molecular dynamics simulations.^71^ These ions break inversion symmetry, and give rise to SFG-active water molecules interacting with the ions, even though they are present in the subsurface region, and not on the surface of water. As shown in Figure 3, ionic solutes such as halides (A) and concentrated hydroxide solutions (B) both lead to dramatic changes in the bonded OH region of the SFG spectrum, including spectral redshifts and broadening below 3400 cm^–1^. These spectral features serve as reliable indicators of perturbations to the local OH environment and heterogeneity. In related work, Ohno et al. model the SFG signal combining χ^(2)^ and χ^(3)^ responses. Consistent with our results, they show that charge densities equivalent to less than 0.005% of a monolayer of water molecules and corresponding to +3 mV led to a detectable difference in the OH stretching region of the SFG spectra.^43^ For sufficiently high ionic strength, we can identify the strong hydrogen bonding interaction associated with high field strengths around small ions with a large charge density, such as F^–^ and OH^–^. Conversely, iodide is less surface active yet still results in significant changes in the hydrogen-bonded region of the spectrum.^8,72^ We include electrolyte solutions here as a benchmark, since ions such as F^–^ and OH^–^ create strong local field effects that produce clear redshifts and broadening of the OH stretch; the absence of such effects in neat water underscores that exceptionally strong interfacial fields are not present at the air–water interface.

**Figure 3:**
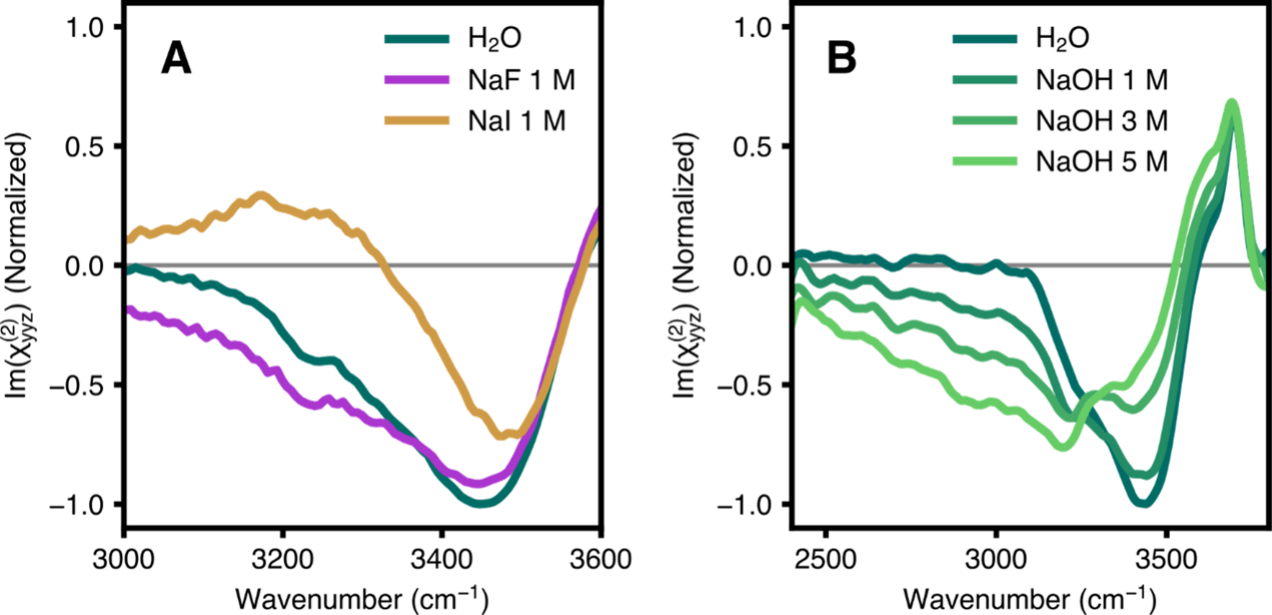
(A) The Imχ^(2)^_*yyz*_ spectra of neat water and aqueous salt solutions (1 M NaF and NaI) at the air–water interface, showing ion-specific perturbations to the bonded OH stretch region. (B) The Imχ^(2)^_*yyz*_ spectra of neat water and aqueous NaOH at increasing concentrations. Hydroxide enrichment at the interface leads to progressive broadening and intensity redistribution in the bonded OH region, producing a low-frequency continuum. Adapted from Litman, Y.; Chiang, K.-Y.; Seki, T.; Nagata, Y.; Bonn, M. Nat. Chem. 2024, 16, 644–650. Available under a CC BY 4.0 license. Copyright 2024 The Author(s).^8^

### Time-Resolved Spectroscopy and Long-Lived Fields

A proposed explanation for enhanced interfacial reactivity is that electric fields at the air–water interface fluctuate more slowly than in bulk, leading to long-lived high-field configurations that could transiently reshape the potential energy surface and promote chemical reactions.^34^ While not involving radicals, there is theoretical evidence that rare, transient field conditions are responsible for the autoionization of water,^73^ where the subsequent ions are seen as important species for surface radical formation.^18,19,33,39,74^ Therefore, multidimensional spectroscopy should be employed to assess the lifetimes of different microscopic structural and energy-redistribution processes, comparing these phenomena in the bulk and at the interface. In the following, we consider vibrational and orientational fluctuations. For aqueous bulk and interfaces, two-dimensional infrared (2D IR) and 2D sum-frequency generation (2D SFG) spectroscopy can directly probe the time scales over which vibrational frequencies fluctuate, a process known as spectral diffusion. Specifically, perturbations to the potential energy surface of each probed molecule follow stochastic trajectories. The ensemble average of the autocorrelation functions for each 0–1 transition energy trajectory (Figure 4A) manifests as time-dependent broadening features in the 2D spectra. For water vibrations, spectral fluctuations on the time scale of hundreds of femtoseconds to a couple picoseconds are directly probed (this is limited on the lower bound by instrument resolution and by vibrational lifetime on the upper bound). Fluctuations faster than hundreds of femtoseconds manifest as homogeneous broadening, and fluctuations slower than picoseconds present themselves as quasi-static heterogeneous broadening.^75^ When complemented by molecular dynamics simulations that provide subfemtosecond resolution of local electric field variations, these spectroscopic techniques offer a powerful framework for comparing fluctuation dynamics across bulk and interfacial water.^38^

**Figure 4:**
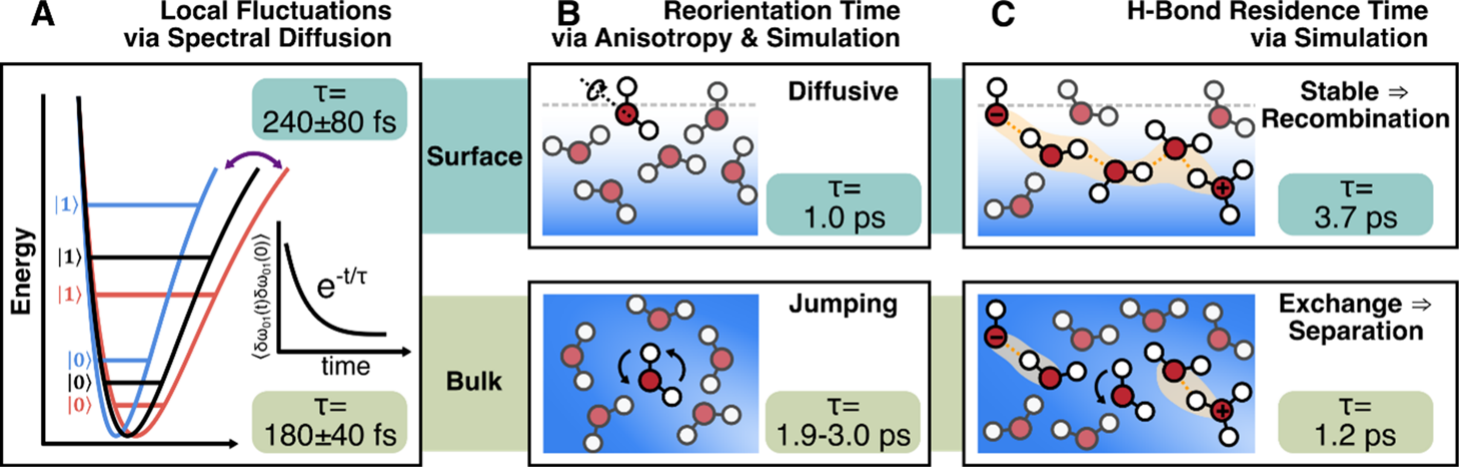
Schematic showing time-dependent differences and similarities between the bulk and surface OH stretches. (A) Spectral diffusion time scales, τ, are similar, indicating no change in the rate the potential energy surface is sampled.^76–79^ (B) Water reorientation follows different mechanisms at the bulk and the interface, resulting in shorter-lived orientations (for the free OH) at the interface.^80–86^ (C) The residence time of hydrogen bonds is longer at the interface, potentially leading to a lower probability of charge separation events.^51,73,87^

In bulk water, 2D IR spectroscopy has shown the frequency fluctuation time constant for the OH stretch to be on the order of 180 ± 40 fs (this is the time associated with the autocorrelation function of the trajectory of the 0–1 transition energy).^76^ Despite some variation in reported values and interpretations, 2D SFG studies by both Bonn and Tahara show that the spectral diffusion of hydrogen-bonded OH modes occurs on similar time scales as the bulk (240 ± 80 fs^77^ and a “few hundred” fs^78^).^79^ These results (Figure 4A) are contrary to the argument for slower interfacial field fluctuations (points 2 and 3, distributions and fluctuations).

In the context of orientational fluctuations, which can be related to reaction coordinate alignment, there are two situations to consider. Namely, fluctuations in alignment with a macroscopic laboratory reference frame, which pertain to static fields, and fluctuations in alignment with a molecular reference frame, which pertain to hydrogen bonding arrangements and other local interactions.

Consider first the macroscopic fields, which may be due to fixed charge gradients or external forces. Dipolar reorientation in this reference frame has been characterized via polarization-dependent anisotropy measurements at the surface and the bulk. Time-resolved SFG coupled with molecular dynamics has shown that the free OH of surface water reorients diffusively with a time scale of around 1.0 ps.^80,81^ In contrast, bulk water has been shown to reorient via a jump mechanism that has a corresponding time scale of 2.2 to 2.5 ps from simulation and 1.9 to 3.0 ps from time-resolved IR spectroscopy (Figure 4B).^82–86^ To our knowledge, the reorientation time scale of the bonded OH at the interface has not been reported.^88^ These data indicate that any molecular alignment with macroscopic fixed fields will be longer-lived in the bulk (points 1 and 3, static e-fields and fluctuations).

Now consider the molecular reference frame; geometric alignment of OH bonds in this frame play a critical role in field-mediated reactivity. For example, the autoionization of water requires a precise sequence of events for the process to occur, which involves the generation of a contact ion pair by rare long-range electrostatic solvent fluctuations, followed by reversible proton transfer through the Grotthuss mechanism (10s of femtoseconds), and finally, stabilization of the separated pair by the breaking of a bridging hydrogen-bond “wire” (picoseconds).^73,87^ Once the ion pair is generated, the slowest and potentially rate limiting step is the rearrangement of the hydrogen bond network, which enables sustained ion separation. Simulations show that the residence time of hydrogen-bonded OH groups is roughly three times longer at the interface than in the bulk (3.7 vs 1.2 ps),^51^ suggesting that recombination of transient ion pairs is more likely at the surface due to hindered network reconfiguration (Figure 4C) (points 2 and 3, field distributions and fluctuations).

### Conclusions

The air–water interface is structurally distinct from the bulk, yet claims that it supports exceptionally large or long-lived electric fields are not substantiated by SFG and time-resolved IR evidence of the O–H stretching region. Surface-specific vibrational spectroscopy, particularly vibrational SFG, reveals no spectral features consistent with the presence of strong interfacial fields. When vibrational frequency-to-field mappings are applied, the resulting field distributions show that stronger electric fields may even be *more* probable in bulk water than at the interface. Our analysis systematically evaluates three proposed mechanisms by which interfacial electric fields might drive enhanced chemical reactivity:

1. Static E-fields: static fields from surface dipoles or charge separation are incompatible with the lack of ion accumulation at the interface,^8^ and there are also no SFG spectral features supportive of a narrowly distributed large field.^59,61,62^

2. Field distributions: local field heterogeneity is present at both surface and bulk, but spectroscopic data show that broader field distributions occur in the bulk.
3. Fluctuations: long-lived field configurations, hypothesized to enable field-aligned reactivity, are contradicted by time-resolved and multidimensional spectroscopy, which demonstrate that the bulk and interface sample energy perturbations occur at a similar rate.^76–79^ Additionally, OH orientations, related to geometric alignment, are longer-lived in the bulk due to differences in reorientation mechanisms.^80–84^ Furthermore, network reorganization occurs faster in the bulk than at the interface, which should facilitate charge separation events.^51,73,87^

These findings indicate that exceptionally large interfacial fields, as sometimes invoked to explain microdroplet reactivity, are not supported by our SFG results. Within the framework of vibrational SFG, we conclude that there is no evidence for such fields at the neat air–water interface. The interpretation of interfacial electric field-driven catalysis in microdroplet experiments must therefore be reconsidered in light of the absence of spectroscopic field signatures at water interfaces. While interfacial environments can modulate orientation and solvation, their electric field strength and longevity do not differ markedly from the bulk in a way that would enhance bond-breaking reactivity, in line with broader redox analyses of peroxide chemistry in aqueous systems.^89^

Enhanced chemistry at interfaces does not require invoking new physics to explain. Several mechanisms to describe the interfacial enhancement have been previously discussed in detail, including altered solvation structure,^36^ confinement effects,^90^ evaporative concentration,^91^ and redox pathways.^92^ In many cases, the observed reactivity likely results from a combination of these factors rather than a single dominant cause. Recent reassessments of droplet reactivity, such as the Diels–Alder reaction, demonstrate that conventional explanations can account for the observed behavior.^90^ Future studies should prioritize time-resolved and multidimensional spectroscopic measurements that directly probe energy fluctuations at interfaces, especially the microdroplet–air interface. Moreover, comparisons of chemical reactivity in matched interfacial and bulk systems under controlled conditions will help disentangle true field effects from structural or kinetic influences. Clarifying the limitations of interfacial electric field strength is essential not only for fundamental surface chemistry but also for atmospheric science, microdroplet catalysis, and the design of water-interface-based technologies.

## Methods

### Application of Vibrational Map

To enable quantitative comparison of electric field distributions from vibrational spectra, the spectral amplitude, Imχ^(n)^, was corrected for the dipole derivative, μ’; polarizability, α_10_; and the displacement of the OH stretch from equilibrium for the 1←0 transition, *x*_10_. This transformation yields an energy distribution, *P* (eq 1). The distribution is calculated using proportionality because it is renormalized before plotting. However, the change in *x*-axis is nonlinear, so the Jacobian, $\left|\frac{d\omega_{10}}{dE}\right|$ , must be applied in order to maintain the probability density. The correction factors were derived from correlations reported by Skinner and co-workers (eq 3–6).^60–62^ These operations were performed across the OH stretching region. Simultaneously, frequency-to-field conversions were performed using the same vibrational electrostatic map employed in the spectral density work (eq 2).^60–62^ The shown frequency-to-field conversion produces two mathematical solutions due to the quadratic dependence of the OH frequency on electric field strength for the given model. Only the positive field solution was retained, as it corresponds to physically meaningful field orientations. This extraneous field rejection was cross-validated against results from a linear mapping models,^47,93^ which are included along with a range of other models the Supporting Information in Figure S1.^44,45,47,60,93^ These calculations yield electric fields in atomic units (a.u.), which are then converted to MV/cm.

$$P\left(E\right)\propto \frac{Im\left(\chi^{\left(n\right)}\right)}{\mu^{\prime }(E)\times x_{10}(\omega_{10})\times \alpha_{10}\left(E,\omega_{10}\right)}\times \left|\frac{d\omega_{10}}{dE}\right|$$ 1

$$\omega_{10}=3761.6\;cm^{-1}+\left(\frac{-5060.4\;cm^{-1}}{a.u.}\times E\right)+\left(\frac{-86225\;cm^{-1}}{a.u.^{2}}\times E^{2}\right)$$ 2

$$\mu^{\prime }\left(E\right)\propto 0.71116+75.591a.u.^{-1}\times E$$ 3

$$x_{10}\left(\omega_{10}\right)=0.1024Å-0.927\times 10^{-5}\;Å\,\;cm^{-1}\times \omega_{10}$$ 4

$$\alpha_{10}\left(E,\omega_{10}\right)\propto x_{10}(\omega_{10})\times \left(1.2142+3.6206\;a.u.^{-1}\times E\right)$$ 5

$$\left|\frac{dE}{d\omega_{10}}\right|=\left|{\left({\left(-5060.4\right)}^{2}-4\times (-86225)\times \left(3761.6-\omega_{10}\right)\right)}^{-1/2}\right|\frac{a.u.}{cm^{-1}}$$ 6

## Experimental Methods

Vibrational sum-frequency generation (SFG) spectroscopy was used to probe the OH stretch region of interfacial water. To eliminate nonresonant background contributions and directly access the molecular absorptive response, the imaginary component of the second-order susceptibility, Imχ^(2)^ was previously measured using heterodyne-detected SFG.^57^ In addition to isolating the resonant vibrational features, Imχ^(2)^ carries orientational information: a negative sign corresponds to OH transition dipoles preferentially oriented into the bulk, while a positive sign indicates dipoles pointing toward the vapor phase. Bulk water responses were obtained by extracting the χ^(3)^ contribution from difference spectra measured using different ion concentrations, as described previously.^11,42^ This χ^(3)^ signal serves as a field-sensitive analogue of the interfacial χ^(2)^ response and allows direct comparison between surface and bulk environments.

To minimize spectral complications from intramolecular coupling, measurements were performed on isotopically diluted HOD in D_2_O (H:D = 1:4). In this system, the OH stretch is spectrally isolated, and its response can be interpreted as arising from two dominant populations. The high-frequency positive feature near 3700 cm^–1^ corresponds to weakly hydrogen-bonded OH groups pointing toward the vapor phase, while the broad negative band centered near 3200–3400 cm^–1^ arises from strongly hydrogen-bonded OH groups oriented toward the liquid. The χ^(3)^ spectrum exhibits a similarly broad feature corresponding to the same frequency range as the bonded OH population. The Imχ^(2)^_*yyz*_ spectra presented in Figures 2 and in [Fig fig3] were obtained from previous work.^8,59^ The Imχ^(3)^_*yyzz*_ spectrum presented in Figure 2 was obtained by measuring the DPPG-water interface at different NaCl concentrations and then taking the subsequent differential spectra (ΔImχ^(2)^_*yyz*_ = Imχ^(2)^_*yyz*,100 mM_–Imχ^(2)^_*yyz*,1 mM_). The details of the experimental spectrum used for the Imχ^(3)^_*yyzz*_ are discussed below.

The experimental SFG spectroscopy instrument uses a 1030 nm fiber laser source (Pharos, Light Conversion). The source is split into two beams. One beam is spectrally narrowed (7 cm^–1^) and used as the up-conversion beam. The second is used to drive a bespoke OPA-DFG system and generate mid-infrared light with a bandwidth (1/e^2^) of ∼800 cm^–1^, yielding spectra from 3000 cm^–1^ to 4000 cm^–1^. The beams are combined collinearly and propagate at an angle of 53°, focusing onto a quartz local oscillator (LO) and then the sample. Between the local oscillator and sample is a 5 mm thick SrTiO_3_ window used to create a 2.1 ps delay between the signal and LO response. The interference pattern is measured using an Andor Kymera 328i spectrograph and Andor iDus 420 CCD cooled to −100 °C. The measurements were performed in the *ssp* polarization combination; respectively, the SFG and visible beams are in the *s* polarization at the plane of incidence of the sample and the MIR beam is in the *p* polarization. At the sample position, the pulse energies are 1.5 μJ for the 1030 nm beam and 0.5 μJ for the mid-IR, with beam diameters of 80 and 150 μm, respectively. Experiments were conducted at a repetition rate of 50 kHz, and the sample trough was rotated to mitigate potential heating effects. Note that previous studies using higher repetition rates and slightly higher pulse energies have reported no measurable heating effects.^94,95^ We also note that we observe no spectral changes using different up-conversion wavelengths at very different repetition rates (Figure S2). More details of the spectroscopic system will be disclosed in a forthcoming publication.

The infrared and Raman spectra of isotopically diluted water were measured using a Bruker A225/Q Platinum ATR accessory with a Bruker Tensor II FTIR spectrometer, and a 532 nm WITec alpha300R Raman microscope equipped with a WITec UHTS 300 spectrometer, respectively.

## Supplementary Material



## Notes

Vibrational map parameters from select publications, a comparison of bulk vs interfacial field strengths at various levels of theory, a detailed discussion about Fresnel factor calculations, a comparison of the air–water SFG spectra at different up-conversion wavelengths; Additional references have been cited in^10,41,44,45,47,60,63,84,93,96–107^ (PDF)
