# Supplementary material for: Reevaluating Anomalous Electric Fields at the Air–Water Interface: A Surface-Specific Spectroscopic Survey
Source: J Am Chem Soc. 2025 Dec 3;147(50):46163–73. doi: 10.1021/jacs.5c14541 (PMC12715790; doi:10.1021/jacs.5c14541)
Supplement: Supplementary file 1 [file ja5c14541_si_001.pdf]

Reevaluating Anomalous Electric Fields at the Air-Water Interface: A Surface-Specific Spectroscopic Survey

Joseph C. Shirley,[1] Zi Xuan Ng,[1] Kuo-Yang Chiang,[1] Yuki Nagata,[1] Yair Litman,[1] Arsh S. Hazrah,[1] Mischa Bonn*[1]*

1. Max Planck Institute for Polymer Research, Ackermannweg 10, 55128 Mainz, Germany

*Corresponding Authors: Mischa Bonn, bonn@mpip-mainz.mpg.de, Arsh S. Hazrah hazraha@mpip-mainz.mpg.de

Electronic Supplementary Information

Contents:

a. **Note S1. Vibrational Maps**
   i. **Table S1**. Vibrational map parameters from select publications
   ii. **Figure S1.** A comparison of bulk vs. interfacial field strengths at various levels of theory
b. **Note S2. Scaling and Fresnel Factor Calculations**
   i. **Figure S2.** A comparison of the air-water spectra with different up-conversion wavelengths
c. **Note S3. Inhomogeneous Broadening Contributions**

Note S1. Vibrational Maps

The vibrational maps for calculating the OH stretching frequency typically follow the form presented in Eq. S1, with ω_{OH} as the calculated frequency, ω_{gas} as the baseline "gas-phase" frequency, a as the coefficient linear with the electric field, E, and b as the coefficient quadratic with the electric field.

$$\omega_{OH} = \omega_{gas} + a * E + b * E^2 \quad \text{Eq. S1}$$

Each of these parameters is given for a range of vibrational maps in Table S1.

Table S1. Vibrational map parameters from select publications.

Model	ω_{gas} (cm^{-1})	a (cm^{-1}/a.u.)	b (cm^{-1}/a.u.^{2})
corcelli_2004_spc[1]	3806	-10792	0
corcelli_2004_tip4p[1]	3832	-12141	0
corcelli_2005[2]	3737	-6932.2	0
auer_2007[3]	3761.6	-5060.4	-86225
gruenbaum_2013[4]	3760.2	-3541.7	-152677
torii_2021[5]	3715.03	-853.4	-203620

The vibrational maps were applied as described in the methods sections of the main text and are plotted in Figure S1. Plot D, titled "auer_2007" is the vibrational map used in the main text.

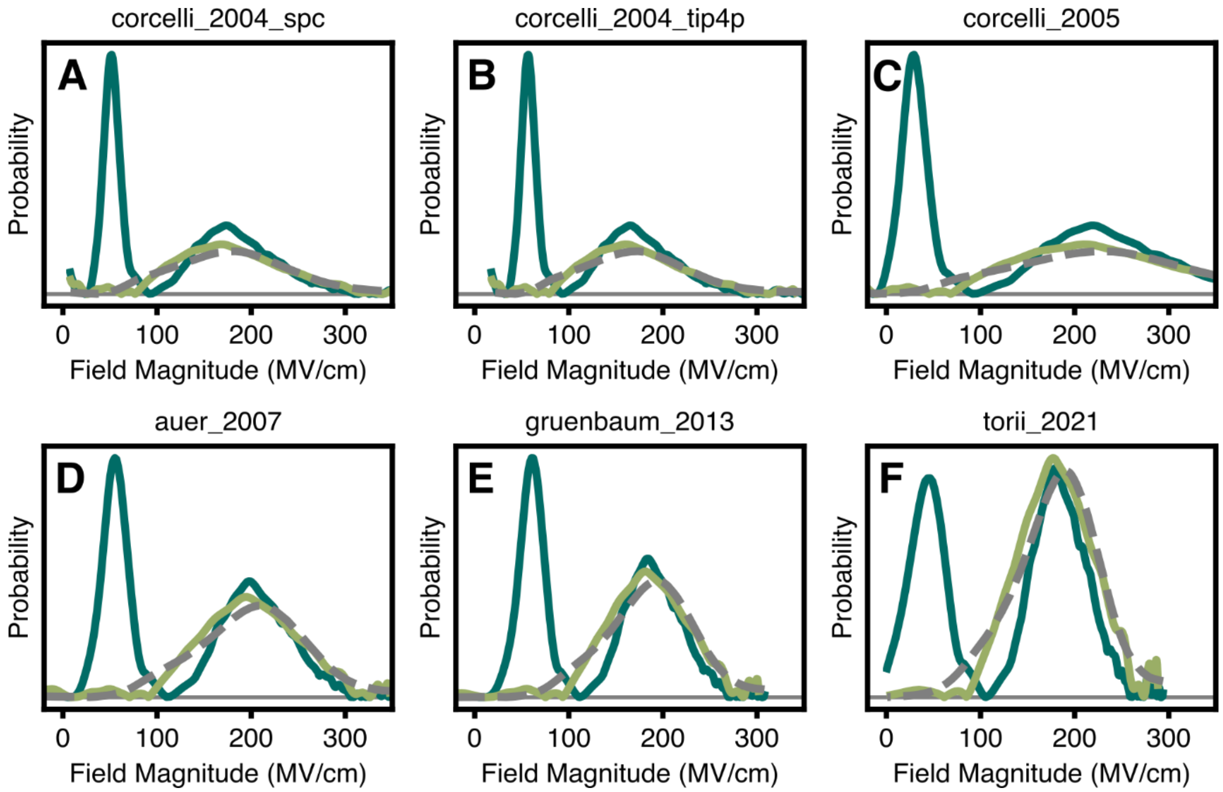

Figure S1. A comparison of bulk vs. interfacial field strengths as in Figure 2A of the main text. In this figure, a variety of vibrational maps are compared. (A) 2004 model derived with the SPC/E forcefield.[1] (B) 2004 derived with the TIP4P forcefield.[1] (C) 2005 model derived with the polarizable SPC-FQ forcefield.[2] (D) 2007 model derived with the SPC/E forcefield.[3] (E) 2013 model improving on previous TIP4P maps.[4] (F) 2021 model based on TIP4P with additional focus on non-electrostatic effects.[5]

Note S2. Scaling and Fresnel Factor Calculations

The reference frame for light in these spectroscopic measurements is the plane of incidence upon the sample, which, for the neat air-water interface, is a $C_{\infty v}$ surface parallel to the laser table. The light polarization *s* is perpendicular to the plane of incidence and the polarization *p* is parallel. In the molecular reference frame, *z* is a vector normal to the sample surface whereas *x* and *y* define

the sample plane. The projection of p is nonzero into x and z and zero into y, and the projection of s into y is unity.[6]

Sum frequency (SFG) wavelengths were read directly from a calibrated spectrometer (Andor Kymera 328i), the wavelength of the upconversion 1030 nm beam was measured with a fiber spectrometer (Avantes, AvaSpec-3648), and the mid-infrared (MIR) wavelengths were calculated as the difference of the frequencies. In this way, the measured discrete array of SFG frequencies are paired with an identically-sized array of MIR frequencies and a singular upconversion frequency. Therefore, many of the values calculated in this section, such as coherence length, are calculated as joint functions of three frequencies (wavelengths). Wavelength-dependent complex refractive indices for air, quartz, and water were obtained for all fundamental, mid infrared, and SFG wavelengths. For air, the complex refractive index was set to 1+0i and not a function of wavelength. Refractive indices for water and quartz were collected from refractiveindex.info,[7] and ultimately from Hale[8] and Franta,[9] respectively. Given the refractive indices of medium I (air) and medium II (water or quartz) the interfacial refractive index was calculated using a slab model ($\phi = \pi$).[10,11] The frequency dependent interfacial refractive index, $n(\omega)_{\text{int}}$, is given by:

$$n(\omega)_{\text{int}} = \sqrt{\frac{1 + (n(\omega)_{mII}^2 - 1) * \frac{2\pi - \phi}{6\pi}}{1 + \frac{1 - n(\omega)_{mII}^2}{6n(\omega)_{mII}^2} * \left(1 + \cos\left(\frac{\phi}{2}\right)\right) * \left(2 - \cos\left(\frac{\phi}{2}\right)\right)}} \qquad \text{Eq. S2}$$

where $n(\omega)_{mII}$ the frequency dependent refractive index of medium II and ϕ is the geometric angle between the surface normal and the line connecting the center of the chromophore sphere to the point of intersection with the dielectric boundary, as defined in Yu et al.[10] In this instance $n(\omega)_{mI}$ is assumed to be one.

The physical geometry of the SFG instrument herein is collinear and has each wavelength propagating at an incident angle of arctan(4/3) (~53°) with respect to the normal of the sample plane. Though the SFG is generated in the sample, we treat it also as a collinear vector with the same incident angles as the MIR and upconversion beam. Therefore, the angle of the beams within medium II is determined with Snell's law.

$$\theta(\omega)_{\mathrm{tran}} = \arcsin\left(\frac{n(\omega)_{mI} * \sin(\theta_{inc})}{n(\omega)_{mII}}\right) \qquad \text{Eq. S3}$$

Where $\theta(\omega)_{\mathrm{tran}}$ is the frequency dependent angle of the transmitted beam, θ_{inc} is the frequency independent angle of incident beam (~53°). $n(\omega)_{mI}$ is set to one.

The propagating frequency dependent wavevectors, $k_z(\omega)$, of each beam, in units of nm^{-1}, can then be calculated as follows with the frequency, ω, in units of cm^{-1}.

$$k_z(\omega) = \frac{1 * 10^{-7}\ \mathrm{cm}}{\mathrm{nm}} * 2\pi\omega * \sqrt{n(\omega)_{mII}^2 - \sin(\theta_{inc})^2} \qquad \text{Eq. S4}$$

Within this framework, the wavevector of the SFG response will be pointing within medium II. Therefore, the coherence length is calculated with addition for this vector. In other literature, the SFG response vector is calculated as the reflection, and therefore must be subtracted to calculate the coherence length. The coherence length, $l_c(\omega)$, is given here as a frequency dependent property. As such, it is critical during this calculation to make sure MIR and SFG frequencies are properly paired.

$$l_c(\omega) = \frac{1}{\left|k_z(\omega)_{MIR} + k_z(\omega)_{up} + k_z(\omega)_{SFG}\right|} \qquad \text{Eq. S5}$$

Here the *MIR, up,* and *SFG* subscripts correspond to the mid-infrared pulse, the up-conversion pulse (1030 nm), and sum frequency generation pulse, respectively.

Once the interfacial refractive indices and transmission angles are calculated, the Fresnel coefficients $(L_{xx}(\omega), L_{yy}(\omega), L_{zz}(\omega))$ can be determined.

$$L_{xx}(\omega) = \frac{2n(\omega)_{mI} * \cos(\theta(\omega)_{tran})}{n(\omega)_{mI} * \cos(\theta(\omega)_{tran}) + n(\omega)_{mII} * \cos(\theta(\omega)_{inc})} \quad \text{Eq. S6}$$

$$L_{yy}(\omega) = \frac{2n(\omega)_{mI} * \cos(\theta(\omega)_{inc})}{n(\omega)_{mI} * \cos(\theta(\omega)_{inc}) + n(\omega)_{mII} * \cos(\theta(\omega)_{tran})} \quad \text{Eq. S7}$$

$$L_{zz}(\omega) = \frac{2n(\omega)_{mII} * \cos(\theta(\omega)_{inc}) * \left(\frac{n(\omega)_{mI}}{n(\omega)_{int}}\right)^2}{n(\omega)_{mI} * \cos(\theta(\omega)_{tran}) + n(\omega)_{mII} * \cos(\theta(\omega)_{inc})} \quad \text{Eq. S8}$$

The Fresnel coefficients are calculated for both the air-water sample interface and the air-quartz reference interface. However, there are also other necessary parameters to quantify the reported spectra. In our heterodyne experiments, we use an LO-before-sample geometry. With this geometry, there is a $-\pi/2$ phase shift that must be applied between the bulk reference and interfacial sample during the referencing process.[12] To scale the spectra, a quantitative value of the $\chi^{(2)}_{ref}$ response for quartz must be used. To our knowledge two main values are used within the SFG community, 6×10^{-13} m/V[13,14] and 8×10^{-13} m/V.[15] We choose here to use the value 6×10^{-13} m/V based on recency and matching the scale of our data with the scales reported in literature.[16] Given these parameters, the *ssp* laboratory-frame data collected from our SFG instrument can first be transformed into *yyz* molecular frame, followed by determination of quantitative units and phase.

$$\chi^{(2)}_{yyz,int,sample} = \frac{\chi^{(2)}_{ssp,int,measured,sample}}{\sigma * \sin(\theta_{inc,MIR}) * L_{yy,SFG}(\omega) * L_{yy,up} * L_{zz}(\omega)_{MIR}} \quad \text{Eq. S9}$$

$$\chi^{(2)}_{yyz,bulk,ref} = \frac{\chi^{(2)}_{ssp,measured,ref}}{\sigma * \cos(\theta_{inc,MIR}) * L_{yy,SFG}(\omega) * L_{yy,up} * L_{xx}(\omega)_{MIR} * l_c(\omega)} \qquad \text{Eq. S10}$$

$$\chi^{(2)}_{yyz,sample} = \frac{\chi^{(2)}_{yyz,int,sample}}{\chi^{(2)}_{yyz,bulk,ref}} * \chi^{(2)}_{ref} * \left(\cos\left(-\frac{\pi}{2}\right) + i\sin\left(-\frac{\pi}{2}\right)\right) \qquad \text{Eq. S11}$$

Here σ is the multiplicity, as described above. $\chi^{(2)}_{yyz,int,sample}$ is the second order nonlinear response arising from the interface of the sample in the molecular frame (unitless). $\chi^{(2)}_{ssp,int,measured,sample}$ and $\chi^{(2)}_{ssp,,measured,ref}$ are the experimentally measured effective nonlinear responses under ssp polarization for the sample and the reference, respectively. $\chi^{(2)}_{yyz,bulk,ref}$ is the second order nonlinear response arising from the bulk of the reference in the molecular frame (unitless). Finally, $\chi^{(2)}_{yyz,sample}$ is the second order nonlinear response of the sample in units of m^2V^{-1}. We emphasize the importance of Fresnel factor–corrected spectra, as they enable direct comparison not only between theory and experiment, but also between measurements obtained from different SFG spectrometers (Figure S2).

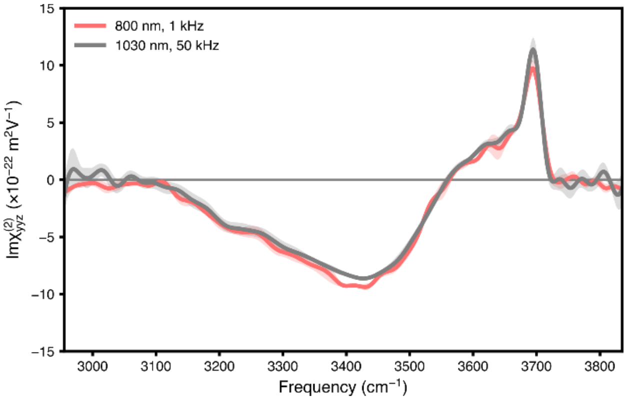

Figure S2. Comparison of the imaginary part of the Fresnel factor–corrected $\chi^{(2)}$ spectra at the air-water interface obtained from two independent SFG spectrometers with different up-conversion wavelengths. The red (grey) curve was measured using a 800 nm (1030 nm) up-conversion pulse at repetition rate of 1 kHz (50 kHz) with an up-conversion pulse energy of 8 μJ (1.5 μJ) and a mid-

infrared pulse energy of 1.5 μJ (0.4 μJ). The excellent agreement between the measurements demonstrates the reproducibility of the heterodyne-detected SFG response across different experimental setups.

Note S3. Estimation of Homogeneous versus Inhomogeneous Broadening Contributions

The measured SFG spectra of the bonded OH stretch reflect a convolution of both homogeneous and inhomogeneous broadening mechanisms. Homogeneous broadening arises from finite vibrational coherence and population lifetimes, whereas inhomogeneous broadening originates from static or slowly varying variations in the local hydrogen-bonding environment. Numerous studies have shown that for liquid water, the bonded OH stretch band is dominated by inhomogeneous broadening, with homogeneous effects contributing only a small fraction of the total linewidth.[17–19] In this analysis, we focus on the bonded OH region rather than the free OH band, as the latter exhibits a narrow, predominantly homogeneous profile with minimal environmental heterogeneity.[18] In contrast, the bonded OH feature is much broader and strongly inhomogeneous, providing a more meaningful measure of the distribution of local hydrogen-bonding configurations and electric fields probed in this work.

To estimate the inhomogeneous component, the total homogeneous dephasing time, T_2, is related to the vibrational population relaxation time, T_1, and the pure dephasing time, T_2^*, as

$$\frac{1}{T_2} = \frac{1}{2T_1} + \frac{1}{T_2^*} \qquad \text{Eq. S12}$$

Here T_1 has been set to 700 fs, as reported in previous studies of HOD in D_2O.[20] The pure dephasing time, T_2^*, was taken as 132 fs based on earlier literature on the same system.[21] Using

these values, the total dephasing time was calculated from Eq. S12 to be T_2= 121 fs. The corresponding homogeneous contribution to the spectral width (in wavenumbers) is then given by

$$\Delta\nu_{Homogeneous} = \frac{1}{\pi c T_2} \qquad \text{Eq. S13}$$

where c is the speed of light ($2.998e10$ cm s^{-1}) and $\Delta\nu_{Homogeneous}$ is the full width at half maximum arising solely form the homogeneous contribution and was determined to be 87 cm^{-1}.

The total observed linewidth can then be expressed as the quadratic sum of homogeneous and inhomogeneous components according to

$$\Delta\nu_{Total}^2 = \Delta\nu_{Inhomogeneous}^2 + \Delta\nu_{Homogeneous}^2 \qquad \text{Eq. S14}$$

where $\Delta\ \nu_{Total}$ is ≈ 300 cm^{-1}. The $\Delta\ \nu_{Inhomogeneous}$ component was determined to be 286 cm^{-1}, which corresponds to roughly 95% of the total contribution.

References

(1) Corcelli, S. A.; Lawrence, C. P.; Skinner, J. L. Combined Electronic Structure/Molecular Dynamics Approach for Ultrafast Infrared Spectroscopy of Dilute HOD in Liquid H2O and D2O. *J. Chem. Phys.* **2004**, *120* (17), 8107–8117. https://doi.org/10.1063/1.1683072.

(2) Corcelli, S. A.; Skinner, J. L. Infrared and Raman Line Shapes of Dilute HOD in Liquid H2O and D2O from 10 to 90 °C. *J. Phys. Chem. A* **2005**, *109* (28), 6154–6165. https://doi.org/10.1021/jp0506540.

(3) Auer, B.; Kumar, R.; Schmidt, J. R.; Skinner, J. L. Hydrogen Bonding and Raman, IR, and 2D-IR Spectroscopy of Dilute HOD in Liquid D2O. *Proc. Natl. Acad. Sci.* **2007**, *104* (36), 14215–14220. https://doi.org/10.1073/pnas.0701482104.

(4) Gruenbaum, S. M.; Tainter, C. J.; Shi, L.; Ni, Y.; Skinner, J. L. Robustness of Frequency, Transition Dipole, and Coupling Maps for Water Vibrational Spectroscopy. *J. Chem. Theory Comput.* **2013**, *9* (7), 3109–3117. https://doi.org/10.1021/ct400292q.

(5) Torii, H.; Ukawa, R. Role of Intermolecular Charge Fluxes in the Hydrogen-Bond-Induced Frequency Shifts of the OH Stretching Mode of Water. *J. Phys. Chem. B* **2021**, *125* (5), 1468–1475. https://doi.org/10.1021/acs.jpcb.0c11461.

(6) Lambert, A. G.; Davies, P. B.; Neivandt, D. J. Implementing the Theory of Sum Frequency Generation Vibrational Spectroscopy: A Tutorial Review. *Appl. Spectrosc. Rev.* **2005**, *40* (2), 103–145. https://doi.org/10.1081/ASR-200038326.

(7) Polyanskiy, M. N. Refractiveindex.Info Database of Optical Constants. *Sci. Data* **2024**, *11* (1), 94. https://doi.org/10.1038/s41597-023-02898-2.

(8) Hale, G. M.; Querry, M. R. Optical Constants of Water in the 200-Nm to 200-Mm Wavelength Region. *Appl. Opt.* **1973**, *12* (3), 555–563. https://doi.org/10.1364/AO.12.000555.

(9) Franta, D.; Nečas, D.; Ohlídal, I.; Giglia, A. Optical Characterization of SiO2 Thin Films Using Universal Dispersion Model over Wide Spectral Range. In *Optical Micro- and Nanometrology VI*; SPIE, 2016; Vol. 9890, pp 253–267. https://doi.org/10.1117/12.2227580.

(10) Yu, C.-C.; Seki, T.; Wang, Y.; Bonn, M.; Nagata, Y. Polarization-Dependent Sum-Frequency Generation Spectroscopy for Ångstrom-Scale Depth Profiling of Molecules at Interfaces. *Phys. Rev. Lett.* **2022**, *128* (22), 226001. https://doi.org/10.1103/PhysRevLett.128.226001.

(11) Yu, X.; Chiang, K.-Y.; Yu, C.-C.; Bonn, M.; Nagata, Y. On the Fresnel Factor Correction of Sum-Frequency Generation Spectra of Interfacial Water. *J. Chem. Phys.* **2023**, *158* (4), 044701. https://doi.org/10.1063/5.0133428.

(12) Nihonyanagi, S.; Mondal, J. A.; Yamaguchi, S.; Tahara, T. Structure and Dynamics of Interfacial Water Studied by Heterodyne-Detected Vibrational Sum-Frequency Generation. *Annu. Rev. Phys. Chem.* **2013**, *64* (Volume 64, 2013), 579–603. https://doi.org/10.1146/annurev-physchem-040412-110138.

(13) Boyd, R. W.; Gaeta, A. L.; Giese, E. Nonlinear Optics. In *Springer Handbook of Atomic, Molecular, and Optical Physics*; Drake, G. W. F., Ed.; Springer International Publishing: Cham, 2023; pp 1097–1110. https://doi.org/10.1007/978-3-030-73893-8_76.

(14) Shoji, I.; Kondo, T.; Kitamoto, A.; Shirane, M.; Ito, R. Absolute Scale of Second-Order Nonlinear-Optical Coefficients. *JOSA B* **1997**, *14* (9), 2268–2294. https://doi.org/10.1364/JOSAB.14.002268.

(15) Wei, X.; Hong, S.-C.; Lvovsky, A. I.; Held, H.; Shen, Y. R. Evaluation of Surface vs Bulk Contributions in Sum-Frequency Vibrational Spectroscopy Using Reflection and Transmission Geometries. *J. Phys. Chem. B* **2000**, *104* (14), 3349–3354. https://doi.org/10.1021/jp9933929.

(16) Fellows, A. P.; Duque, Á. D.; Balos, V.; Lehmann, L.; Netz, R. R.; Wolf, M.; Thämer, M. How Thick Is the Air–Water Interface?─A Direct Experimental Measurement of the Decay Length of the Interfacial Structural Anisotropy. *Langmuir* **2024**, *40* (35), 18760–18772. https://doi.org/10.1021/acs.langmuir.4c02571.

(17) Brünig, F. N.; Geburtig, O.; Canal, A. von; Kappler, J.; Netz, R. R. Time-Dependent Friction Effects on Vibrational Infrared Frequencies and Line Shapes of Liquid Water. *J. Phys. Chem. B* **2022**, *126* (7), 1579–1589. https://doi.org/10.1021/acs.jpcb.1c09481.

(18) Hsieh, C.-S.; Campen, R. K.; Okuno, M.; Backus, E. H. G.; Nagata, Y.; Bonn, M. Mechanism of Vibrational Energy Dissipation of Free OH Groups at the Air–Water Interface. *Proc. Natl. Acad. Sci.* **2013**, *110* (47), 18780–18785. https://doi.org/10.1073/pnas.1314770110.

(19) Auer, B. M.; Skinner, J. L. Vibrational Sum-Frequency Spectroscopy of the Water Liquid/Vapor Interface. *J. Phys. Chem. B* **2009**, *113* (13), 4125–4130. https://doi.org/10.1021/jp806644x.
(20) Fecko, C. J.; Loparo, J. J.; Roberts, S. T.; Tokmakoff, A. Local Hydrogen Bonding Dynamics and Collective Reorganization in Water: Ultrafast Infrared Spectroscopy of HOD/D2O. *J. Chem. Phys.* **2005**, *122* (5), 054506. https://doi.org/10.1063/1.1839179.
(21) Stenger, J.; Madsen, D.; Hamm, P.; Nibbering, E. T. J.; Elsaesser, T. Ultrafast Vibrational Dephasing of Liquid Water. *Phys. Rev. Lett.* **2001**, *87* (2), 027401. https://doi.org/10.1103/PhysRevLett.87.027401.
